# Evaluation of the Association Between Genetic Variants in Circadian Rhythm Genes and Posttraumatic Stress Symptoms Identifies a Potential Functional Allele in the Transcription Factor *TEF*

**DOI:** 10.3389/fpsyt.2018.00597

**Published:** 2018-11-15

**Authors:** Sarah D. Linnstaedt, Yue Pan, Matthew C. Mauck, Jenyth Sullivan, Christine Y. Zhou, Lindsey Jung, Cathleen A. Rueckeis, Jameson D. Blount, Matthew S. Carson, Andrew S. Tungate, Michael C. Kurz, Phyllis L. Hendry, Christopher Lewandowski, Teresa D'Anza, Elizabeth Datner, Kathy Bell, Megan Lechner, Jeffrey W. Shupp, Bruce A. Cairns, Samuel A. McLean

**Affiliations:** ^1^Institute for Trauma Recovery, University of North Carolina, Chapel Hill, NC, United States; ^2^Department of Anesthesiology, University of North Carolina, Chapel Hill, NC, United States; ^3^Department of Biostatistics, University of North Carolina, Chapel Hill, NC, United States; ^4^Department of Biostatistics, Boston University School of Public Health, Boston, MA, United States; ^5^Department of Emergency Medicine, University of Alabama School of Medicine, Birmingham, AL, United States; ^6^Department of Emergency Medicine, University of Florida College of Medicine, Jacksonville, FL, United States; ^7^Department of Emergency Medicine, Henry Ford Hospital, Detroit, MI, United States; ^8^Albuquerque Sexual Assault Nurse Examiner Collaborative, Albuquerque, NM, United States; ^9^Department of Emergency Medicine, Albert Einstein Medical Center, Philadelphia, PA, United States; ^10^Forensic Nursing Program, Tulsa Police Department, Tulsa, OK, United States; ^11^Forensic Nursing Program, Memorial Health System, Colorado Springs, CO, United States; ^12^Department of Surgery, The Burn Center, MedStar Washington Hospital Center, Georgetown University School of Medicine, Washington, DC, United States; ^13^Jaycee Burn Center, University of North Carolina, Chapel Hill, NC, United States; ^14^Department of Emergency Medicine, University of North Carolina, Chapel Hill, NC, United States

**Keywords:** PTSD, genetic polymorphism, circadian rhythm, trauma, TEF, RNA

## Abstract

Previous studies suggest that genetic variants within genes affecting the circadian rhythm influence the development of posttraumatic stress symptoms (PTSS). In the present study, we used data from three emergency care-based cohorts to search genetic variants in circadian pathway genes previously associated with neuropsychiatric disorders for variants that influence PTSS severity. The three cohorts used included a discovery cohort of African American men and women enrolled following motor vehicle collision (*n* = 907) and two replication cohorts: one of multi-ethnic women enrolled following sexual assault (*n* = 274) and one of multi-ethnic men and women enrolled following major thermal burn injury (*n* = 68). DNA and RNA were collected from trauma survivors at the time of initial assessment. Validated questionnaires were used to assess peritraumatic distress severity and to assess PTSS severity 6 weeks, 6 months, and 1 year following trauma exposure. Thirty-one genetic variants from circadian rhythm genes were selected for analyses, and main effect and potential gene^*^stress and gene^*^sex interactions were evaluated. Secondary analyses assessed whether associated genetic variants affected mRNA expression levels. We found that six genetic variants across five circadian rhythm-associated genes predicted PTSS outcomes following motor vehicle collision (*p* < 0.05), but only two of these variants survived adjustment for multiple comparisons (False Discovery Rate < 5%). The strongest of these associations, an interaction between the PAR-zip transcription factor, thyrotroph embryonic factor (*TEF*) variant rs5758324 and peritraumatic distress, predicted PTSS development in all three cohorts. Further analysis of genetic variants in the genetic region surrounding *TEF*rs5758324 (±125,000 nucleotides) indicated that this allele showed the strongest association. Further, *TEF* RNA expression levels (determined via RNA-seq) were positively associated with PTSS severity in distressed individuals with at least one copy of the *TEF*rs5758324 minor allele. These results suggest that rs5758324 genetic variant in *TEF*, a regulator of clock-controlled genes and key mediator of the core circadian rhythm, influence PTSS severity in a stress-dependent manner.

## Introduction

Unfortunately, traumatic events are common in life. For instance, each year in the United States, more than 11 million individuals experience a motor vehicle collision (MVC) (1), more than one million women are sexually assaulted (2), and more than 50,000 individuals are hospitalized after major thermal burn injury (3). Although most individuals recover following trauma exposure, a substantial proportion develop adverse post-traumatic neuropsychiatric sequelae such as persistent posttraumatic stress symptoms (PTSS).

Individual genetic differences influence vulnerability to PTSS following trauma exposure: data from twin research and genetic association studies estimate the heritability of PTSS to be between 29 and 40 percent (4–6). The study of genetic variants associated with vulnerability to PTSS has provided a number of valuable clues and new directions in understanding the pathogenesis and manifestations of the disorder. The first GWAS for PTSS identified an association between genetic variants in the retinoid-related orphan receptor alpha gene, a gene that stabilizes environmental influences on the circadian rhythm (7), and PTSS vulnerability (8). This association has been subsequently replicated in both studies of PTSS (9, 10) and other stress-related disorders (11–17) [but also failed to replicate for PTSS (18)].

In addition to these data, evidence continues to accrue more broadly that the circadian rhythm plays an important role in PTSS development and symptom expression (19–30). The circadian rhythm entrains the body to 24 h light-dark cycles through transcription-translation feedback loops in the hypothalamic suprachiasmatic nucleus and throughout the body in cells of almost every tissue (31, 32). This cycling allows the body to adjust behavior, metabolism, and physiology in response to environmental cues. One specific type of environmental stimulus known to influence the circadian rhythm (and vice versa) is physiological stress exposure (33, 34). It has been hypothesized that interactions between circadian and stress systems may contribute to the underlying pathogenesis of neuropsychiatric disorders (35), and a growing body of evidence supports this hypothesis (36–40).

Evidence supporting the interplay between the circadian clock and stress systems includes data from a variety of studies. For instance, clock genes (e.g., *PER1, PER2, TIMELESS, NPAS2*) have been shown to be regulated by stress hormones such as glucocorticoids (GC) (41–43). GC release throughout a 24 h period follows a circadian rhythm in which levels are highest at the beginning of the wake period and lowest at the beginning of the sleep period (44). Animals with key circadian rhythm genes knocked out have altered GC levels (45). Additionally, FKBP5, a chaperone protein that plays a role in the regulation of circulating GC levels, and has been shown to play a role in stress related disorders (46, 47), is rhythmically expressed in most tissues (48).

In the current study, we used prospective data from three emergency department based trauma cohorts (one discovery and two replication cohorts) to test the hypothesis that genetic variants in circadian rhythm genes predict PTSS development following trauma exposure. In addition, we hypothesized that the relationship between polymorphisms in circadian rhythm genes and PTSS might be dependent on stress levels (as measured via reported peritraumatic distress levels or via the critical stress regulator *FKBP5*). We found a significant, stress-dependent relationship between a genetic variant in the circadian rhythm-associated thyrotroph embryonic factor (*TEF*) gene, rs5758324, and PTSS development that replicated across all three cohorts. In addition, analyses of mRNA expression data from the discovery cohort suggest that this allele is functional.

## Methods

### Study design, setting, and eligibility criteria

#### Motor vehicle collision (MVC) study

This prospective longitudinal study enrolled African American individuals ≥18 and ≤65 years of age presenting to the ED within 24 h of MVC. The details of this study have been described previously (49). This study was approved by the institutional review boards at the data-coordinating center (The University of North Carolina at Chapel Hill) and at all participating hospitals. Each study participant provided written informed consent before enrollment.

Eligible and consenting participants provided blood samples in the ED and completed an ED interview evaluation. Research assistants performed interview evaluations at the time of the ED visit using a web-based survey with explicit definitions of variables. Patients who were not alert and oriented were excluded, as were patients who did not self-identify as African American, pregnant patients, prisoners, patients unable to read and understand English, or patients taking opioids above a total daily dose of 30 mg of oral morphine or equivalent. Data extraction from the ED medical record obtained injury characteristics and medications administered in the ED. Participant demographic characteristics (including age, sex, and educational attainment) were obtained from the ED medical record and from participant self-report. Six weeks, six months, and one year after the MVC, participants completed a follow-up interview by telephone, online, or via mail.

##### Assessments and outcome definitions for the MVC study

MVC-related PTSS was assessed at all three follow-up timepoints (6 weeks, 6 months, and 1 year) using the Impact of Event Scale: Revised (IESR) (50). This 22-item questionnaire includes avoidance, intrusion and hyperarousal subscales. Scores range from 0 to 88. Using a previously validated cutoff [IESR ≥ 33 (51)], we estimated that 29% of individuals enrolled following MVC had PTSD 6 months following trauma exposure.

Peritraumatic distress in the ED was measured using the Peritraumatic Distress Inventory, a 13-item questionnaire assessing the level of distress experienced immediately after a traumatic event (52). This assessment measures life threat, loss of control, helplessness/anger, and guilt/shame. Each item on the questionnaire was evaluated via numeric rating scale from 0 (no distress) to 4 (high distress). A validated cut-off score of 23 was used to identify those with substantial distress (53).

#### Sexual assault (SA) study

This prospective longitudinal study is similar in design to its pilot study described previously (54) and to the MVC study described above. Women ≥18 and ≤65 years of age presenting to one of 13 sexual assault nurse examiner (SANE) programs within 72 h of sexual assault trauma were enrolled. Women unable to give informed consent (e.g., due to intoxication) were excluded, as were women who were hospitalized after sexual assault, lived with their assailant, were prisoners, were pregnant, did not have a telephone, and/or did not live within driving distance for follow-up interviews. Institutional Review Board (IRB) approval was obtained at all study sites, and all study participants provided written informed consent.

##### Assessments and outcome definitions for the SA study

SA-related PTSS was assessed at three follow-up timepoints (6 weeks, 6 months, and 1 year) using the civilian version of the posttraumatic stress disorder checklist (PCL) (55). This validated 17-item questionnaire assesses posttraumatic distress symptoms in relation to a stressful experience. A total symptom severity score (range = 17–85) can be obtained by summing the scores from each of the 17 items that have response options ranging from 1 “Not at all” to 5 “Extremely.” Using this PCL scale, we estimated the incidence of PTSD 6 months following trauma exposure in this cohort of sexual assault survivors to be 62%.

One week following sexual assault, participants were asked about peritraumatic distress symptoms in the period since the assault using measures from PROMIS 8b. These measures include statements such as “I felt fearful,” “I felt nervous,” “I felt anxious,” “I felt tense,” and “I felt uneasy.” Participants rated each item with a score from 1 to 5 (ranging from never to always), with a total possible score from 8 to 40. The median score was used to distinguish women with high distress from those with low distress.

#### Major thermal burn injury (MThBI) study

Patients undergoing tissue autograft after MThBI between February 2012 through June 2015 at one of the three burn centers (University of North Carolina, Chapel Hill, NC, MedStar Washington Hospital Center, Washington, DC, and University of South Florida, Tampa, FL) were enrolled. Exclusion criteria included age <18 or >65, admission >72 h after MThBI, estimated total body surface area (TBSA) burn >30%, intentional, electrical or a chemical mechanism, autograft performed >14 days after admission to burn center or autograft decision made >7 days after admission, Childs-Pugh liver failure stage B or C, end stage renal disease, chronic opioid use >20 morphine equivalents per day before burn, preburn skin disorder causing pruritus, substantial co-morbid injury (e.g., blast injury resulting in major trauma in addition to burn), pregnancy or breastfeeding, residing greater than 100 miles from site, and burn that required escharotomy. In addition, individuals unwilling to provide a blood sample, prisoners, suicidal, homicidal, psychotic individuals, and individuals who did not read and speak English were excluded. The Institutional Review Board at each burn center approved the study protocol, and each participant provided written informed consent.

##### Assessments and outcome definitions for the MThBI study

MThBI-related PTSS severity was assessed 1 day, 6 weeks, 6 months, and 1 year following burn injury using the PCL questionnaire (as described above for the SA study). In this MThBI cohort, an estimated 13% of individuals reported PTSD 6 months following trauma exposure. This questionnaire was also administered in the immediate aftermath of trauma (day 1) and served as the measure for peritraumatic distress. A previously reported cutoff of 23 was used to distinguish individuals with high distress vs. those with low distress (56).

### DNA collection and genotyping

Study personnel collected blood samples at the time of enrollment using PAXgene DNA tubes. Following DNA purification (PAXgene blood DNA kit, QIAGEN), genotyping using the Infinium Multi-Ethnic Global Array (MEGA, Illumina) was performed (AKESOgen, Inc; Atlanta, GA). DNA from an individual with known genotype (NA19819, Coriell Institute, Camden, NJ) and two repeat samples were included in each genotyping batch (96 samples) to ensure genotypic accuracy and reliability.

Thirty-one genetic variants across nine circadian rhythm associated genes were selected for analyses based on previous association with PTSS or related neuropsychiatric disorders (see Table 1 for references) and whether they were included on the MEGA chip. These genetic variants had excellent call rates (>98%) and were in Hardy-Weinberg equilibrium (Supplementary Table 1, *p* ≤ 0.05).

**Table 1:** Baseline characteristics of study participants.

	**Discovery**	**Replication cohorts**	
**Characteristic**	**MVC^a^**	**SA^b^**	**MThBI^c^**
Enrolled, n	930	274	68
Age, years, mean (SD)	35.1 (12.7)	28.8 (11.4)	37.6 (12.2)
Females, *n* (%)	578 (62.2)	274 (100)	18 (26.5)
African American, *n* (%)	930 (100)	46 (16.8)	27 (39.7)
Education, *n* (%)			
8–11 yrs	71 (7.6)	17 (6.2)	8 (11.8)
High school	290 (31.2)	70 (25.5)	24 (35.3)
Post-high school	42 (4.5)	12 (4.4)	2 (2.9)
Some college	338 (36.3)	124 (45.3)	23 (33.8)
College graduate	137 (14.7)	44 (16.1)	8 (11.8)
Post-graduate studies	36 (3.9)	7 (2.6)	3 (4.4)

a MVC, motor vehicle collision cohort;

b SA, sexual assault cohort;

c *MThBI, major thermal burn injury cohort*.

### RNA collection and sequencing

PAXgene RNA tubes were used to collect blood in the ED at the time of enrollment. Total RNA was isolated using the PAXgene blood miRNA kit (QIAGEN) and stored at −80°C until use. RNA concentration and purity were measured using a NanoDrop 1000 (Nanodrop Technologies, Wilmington, DE).

One hundred and eighty-four samples were selected for RNA sequencing based on whether individuals reported relatively high or relatively low levels of PTSS over time after MVC. This selection occurred intermittently throughout the study and well prior to the planning of these specific analyses.

Template libraries for total RNA sequencing were produced from 600 ng total RNA using Ovation Human Blood RNA-Seq Library Systems kit (NuGen, San Carlos, CA) according to manufacturer's specifications. Libraries were multiplexed in groups of six and sequenced on a HiSeq 2500 at the University of North Carolina at Chapel Hill High Throughput Sequencing Facility. Raw sequencing reads were aligned to the human hg19 genome assembly using STAR (version 2.4.2a) (57). Expression levels of each transcript (*n* = 20,353) were estimated via RSEM (58) using University of California Santa Cruz (UCSC) known gene transcript and gene definitions. Raw RSEM read counts for all samples were normalized to the overall upper quartile (59) before comparison and visualization. Consistent with study goals, only messenger RNA aligning to the *TEF* gene was included in these analyses.

### Selection of circadian rhythm associated genetic variants for primary analyses

The genetic variants selected for analyses in this study originate from genes in the core circadian rhythm pathway that are involved directly in the core feedback mechanism or are transcriptional regulators of clock-controlled genes that affect tissue specific physiological processes such as neurotransmission, immune processes, and endocrine signaling (Supplementary Figure 1). We used a structured literature review to identify genetic variants within such circadian rhythm genes that have previously been shown to be associated with PTSS or related neuropsychiatric disorders. To perform this literature review, we first searched the PubMed (NCBI) database using the following search terms: “post-traumatic stress disorder”/“PTSD” and “*CLOCK*” or “*BMAL1*” or “*BMAL2*” or “*ARNTL*” or “*ARNTL2*” or “*PER1*” or “*PER2*” or “*PER3*” or “*CRY1*” or “*CRY2*” or “*REV-ERB*” or “*NR1D2*” or “*NR1D1*” or “*RORA*” or “*RORB*” or “*CSNK1E*” or “*NPAS2*” or “*TEF*” or “*TIMELESS*” or “*VIP*,” “*VIPR2*.” We then searched for related neuropsychiatric disorders as the outcome in conjunction with each of the genes listed above. This search resulted in 207 total manuscripts describing 204 genetic variants with association to the queried disorders (Supplementary File 1). We then grouped these genetic variants into four tiers based on the following criteria: *Tier 1*: significant association between the genetic variant and the above defined neuropsychiatric disorders across two or more previous reports, and minor allele frequency (MAF > 0.05) (*n* = 19 genetic variants); *Tier 2*: significant association between the genetic variant and the above defined neuropsychiatric disorders in at least one previous report, and MAF > 0.05 (*n* = 98 genetic variants); *Tier 3*: no significant association detected in previous report(s) (*n* = 24 genetic variants); *Tier 4*: MAF < 0.05 or no record of the MAF for African or Caucasian populations (*n* = 63 genetic variants). We then selected genetic variants from Tiers 1 and 2 for association analyses (*n* = 117 genetic variants). However, only a subset of these genetic variants were included on the genotyping array we had used to assess genetic variants in these cohorts. Thus, thirty-one genetic variants across nine circadian rhythm associated genes were included in primary analyses (Supplementary File 1 and Supplementary Table 2).

### Analyses

#### Genetic association analyses

Sociodemographic characteristics of the sample were summarized using standard descriptive statistics. Repeated measures mixed models were used to evaluate the association between each of the 31 genetic variants and PTSS outcomes over time and for the following potential interactions: sex × genetic variant, peritraumatic distress × genetic variant, and *FKBP5* (using the tagging allele, rs3800383) × genetic variant. [*FKBP5* was included in these analyses as a secondary measure for stress interactions, since *FKBP5* is a critical mediator of the hypothalamic pituitary axis and is highly associated with PTSS (46)]. Models were adjusted for potential confounding by age, education level, time since the traumatic event, and enrollment study site. Sex, distress, and *FKBP5*-dependent effects were evaluated because of increasing evidence that such interactions are frequently present [e.g., (60–62)] and because such effects have been found for circadian rhythm associated genes previously (36–40). A dominant genetic model [two copies of the major allele (coded as 0) vs. one or more copies of the minor allele (coded as 1)] was used for all models. Marginal means corresponding to PTSS severity were derived from the fully adjusted models. False discovery rate (FDR) was controlled for in each analysis subset (e.g., main effects, stress interaction, sex interaction) separately. when determining statistical significance. All analyses were performed using SPSS and SAS software (v24; SPSS Inc. Chicago, IL; SAS 9.4, SAS Institute Inc., Cary, NC). Of note, the raw data supporting the conclusions of this manuscript will be made available by the authors, without undue reservation, to any qualified researcher.

#### RNA expression analyses

RNA expression data was only available for the MVC cohort; thus analyses assessing the relationship between the genetic variant *TEF*rs5758324 and its effect on *TEF* mRNA expression were assessed in this cohort alone. The total cohort with RNA data (*n* = 184) was stratified by high and low distress and by rs5758324 major vs. minor allele. For each subgroup, bivariate analyses were used to assess the strength and direction of the relationship between *TEF* mRNA expression and PTSS severity. Due to non-normal distribution of *TEF* mRNA sequencing reads (Shapiro-Wilk test for normality, *p* < 0.05), Spearman's rank correlation coefficients were reported.

#### Bioinformatics analyses

Linkage disequilibrium was assessed in this study using LDlink, a web-based application that enables exploration of population specific linkage structures based on data from 1000 Genomes Project Phase 3 and dbSNP build 142 (63).

## Results

### Participants

Baseline characteristics of participants in the discovery and replication cohorts are shown in Table 1. The motor vehicle collision (MVC) cohort has been described previously (49). This study exclusively enrolled African American women and men (*n* = 930) who reported to the emergency department (ED) within 24 h of an MVC. The sexual assault (SA study) is an on-going study of multi-ethnic women who presented for emergency care within 72 h of SA. All participants enrolled in the SA study with PTSS outcome data available at the time of analyses were genotyped (*n* = 274). The major thermal burn injury (MThBI) study enrolled African American and European American women and men within 72 h of thermal burn injury (*n* = 68). Follow-up rates at the final 1 year follow-up timepoint were ≥ 85% for all three studies.

### Genetic variants selected for primary analyses

Because our cohort sizes were relatively small, to reduce the likelihood of type I and type II error we performed a candidate gene study using genetic variants in circadian rhythm associated genes previously associated with neuropsychiatric disorder vulnerability. These 31 genetic variants (Table 2), were identified via structured literature review (see Methods for details).

**Table 2:** Genetic variants in circadian rhythm genes that have previously been shown to be associated with neuropsychiatric disorders and are the focus of primary analyses in the current study (*n* = 31 genetic variants).

**Gene name**	**Gene symbol**	**Genomic location**	**SNP**	**Previous associations**
Aryl hydrocarbon receptor nuclear translocator like	*ARNTL; BMAL1*	11p15	rs7107287	Anxious temperament (64); BD (65)
			rs1982350	Depression (66); BD (65)
			rs11022778	MDD, appetite changes (67); age at first suicide attempt (68)
			rs969485	Depression (66)
Clock circadian regulator	*CLOCK*	4q12	rs534654	Violent suicide attempts (68); BD (69)
			rs1801260	MDD in males (36); BD (70, 71); SAD (72); appetite disturbance (67); MDD (73); chronotype (74); evening activity (75, 76); insomnia during depression treatment (77); insomnia with MDD + BD (78)
Neuronal PAS domain protein 2	*NPAS2*	2q11.2	rs1562313	BD with seasonal pattern (79)
			rs12622050	BD with seasonal pattern (79)
			rs2305159	BD with seasonal pattern (79)
			rs6740935	MDD (73)
Period 2	*PER2*	2q37.3	rs6431590	MDD (17)
Period 3	*PER3*	1p36.23	rs10462018	MDD (73)
			rs228642	MDD (80)
RAR related orphan receptor A	*RORA*	15q22.2	rs4774388	Depression (66); BD (81)
			rs2414680	MDD (73)
			rs16943472	MDD (73)
			rs4775351	MDD (73)
			rs8023563	Depression (16)
			rs12906588	Depression (16)
			rs809736	Response to antidepressants (15)
			rs782931	BD (82)
			rs13329238	BD (81)
			rs9302215	BD (81)
			rs11071557	BD (81)
			rs12915776	BD (81)
			rs8041466	BD (81)
			rs34720147	BD (81)
RAR related orphan receptor B	*RORB*	9q22	rs7022435	BD (83)
TEF, PAR bZIP transcription factor	*TEF*	22q13.2	rs738499	Depression (74, 84–87); sleep disturbances (88)
			rs5758324	MDD (73)
Timeless circadian clock	*TIMELESS*	12q13.3	rs11171856	violent suicide attempts (68)

*MDD, major depressive disorder; BD, bipolar disorder*.

### Relationship between circadian rhythm associated genetic variants and PTSS severity following trauma exposure

#### MVC discovery cohort analyses

Repeated measures mixed models adjusting for ED study site, participant age, education level, and sex were used to assess whether any of the 31 variants identified in previous literature predicted PTSS severity after MVC. For each genetic variant, we assessed for main effect relationships with PTSS and for potential interactions with sex, peritraumatic distress, and *FKBP5* (using the rs3800373 tagging allele). Eight associations were identified (Table 3)). Two of these, from the thyrotroph embryonic factor (*TEF*) gene, survived false discovery rate (FDR < 0.05) adjustment: *TEF*rs5758324^*^peritraumatic distress [*F*_(1, 824)_ = 10.54, *p* = 3.7 × 10^−2^] and *TEF*rs738499^*^FKBP5 [*F*_(1, 830)_ = 10.05, *p* = 4.96 × 10^−2^]. Stratified analyses demonstrating the direction and magnitude of effect for all associations in Table 3) are presented in Supplementary Figure 2). Additionally, demographic data stratified by allele for *TEF*rs5758324 is presented in Supplementary Table 3).

**Table 3:** Relationship between genetic variants in circadian rhythm genes and posttraumatic stress symptom severity following motor vehicle collision trauma in African American individuals (*n* = 930).

**Gene name**	**SNP**	**Alleles**	**Interaction**	**F statistic**	*p*-value^*^	***p*-value^#^ (FDR adj.)**
*RORB*	rs7022435	G/A	-	5.22	2.26 ^*^ 10^−2^	0.315
*BMAL1*	rs969485	A/G	-	5.13	2.38 ^*^ 10^−2^	0.315
*RORA*	rs4774388	T/C	-	4.70	3.05 ^*^ 10^−2^	0.315
*RORA*	rs4774388	T/C	sex	6.18	1.31 ^*^ 10^−2^	0.406
*NPAS2*	rs12622050	G/A	distress	5.10	2.41 ^*^ 10^−2^	0.250
*TEF*	rs5758324	T/G	distress	10.54	1.20 ^*^ 10^−3^	**3.70** ^*^10^−2^
*TEF*	rs738499	T/G	distress	8.69	3.30 ^*^ 10^−3^	5.10^*^10^−2^
*TEF*	rs738499	T/G	*FKBP5*	10.05	1.60 ^*^ 10^−3^	**4.96** ^*^10^−2^

Relationships examined include genetic variant main effects, and interactions between the genetic variant and participant sex, peritraumatic distress, or FKBP5 tagging allele, rs3800373.

* *p-value generated via repeated measures mixed models (6 week, 6 months, 1 year), adjusted for age, sex, emergency department enrollment site, education, and time following motor vehicle collision. A dominant genetic model was used for all genetic polymorphisms*.

# *False discovery rate (FDR) adjusted p-values*.

*Bold values indicate significance at the p < 0.05 threshold*.

#### Replication analyses in SA and MThBI cohorts

In repeated measures mixed model replication analyses, *TEF*rs5758324 significantly predicted PTSS severity in a peritraumatic distress-dependent manner in both the SA cohort [*F*_(1, 257)_ = 5.52, *p* = 0.020, Table 4] and the MThBI cohort [*F*_(1, 57)_ = 5.72, *p* = 0.020, Table 4]. Because the discovery cohort was comprised of only African American individuals (MAF = 0.35), we also assessed whether *TEF*rs5758324 predicted PTSS when limiting analyses to this strata alone (16.8% of SA cohort, 39.7% of MThBI cohort) (MAF range for different ancestry groups = 0.17–0.78; overall MAF = 0.48). We observed similar results for African American individuals as for the full cohort [SA, *F*_(1, 33)_ = 4.57, *p* = 0.040; MThBI, *F*_(1, 18)_ = 7.52, *p* = 0.013].

**Table 4:** Relationship between genetic variants in circadian rhythm genes and posttraumatic stress symptoms in individuals following sexual assault (SA, *n* = 274) and major thermal burn injury (MThBI, *n* = 68) trauma.

			**SA cohort**		**MThBI cohort**	
**Gene name**	**SNP**	**Interaction**	*p*-value (All)^*^	*p*-value (AA)^*^	*p*-value (All)^*^	*p*-value (AA)^*^
*RORB*	rs7022435	–	0.641	0.112	*NA*	*NA*
*BMAL1*	rs969485	–	0.663	0.546	0.351	0.443
*RORA*	rs4774388	–	0.579	0.630	0.601	0.928
*RORA*	rs4774388	sex	–	–	0.505	0.899
*NPAS2*	rs12622050	distress	0.171	0.131	0.296	0.333
*TEF*	rs5758324	distress	**0.020**	**0.040**	**0.020**	**0.013**
*TEF*	rs738499	distress	0.233	0.421	0.524	0.206
*TEF*	rs738499	*FKBP5*	0.995	0.665	0.485	0.909

*Only genetic variants shown to be associated with posttraumatic stress symptoms following motor vehicle collision (MVC) were assessed in these cohorts*.

* *p-value generated via repeated measures mixed models (6 week, 6 months, 1 year), adjusted for age, sex, emergency department enrollment site, education, and time following sexual assault (SA) or major thermal burn injury (MThBI). AA, African American individuals only; NA, genetic variant not available*.

*Bold values indicate significance at the p < 0.05 threshold*.

### *TEF*rs5758324 predicts PTSS severity following trauma exposure in a stress dependent manner

**Figure 1:**
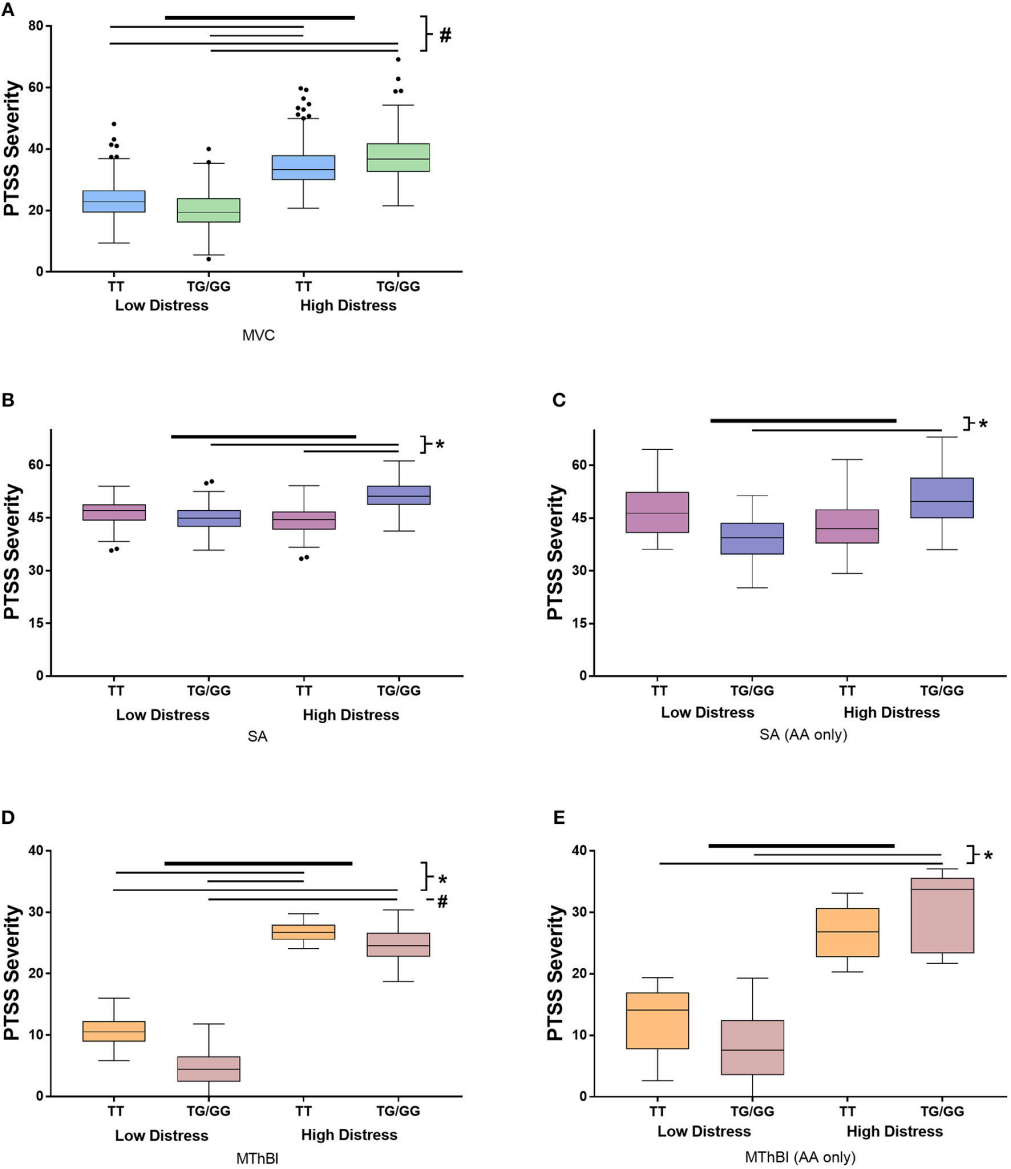
Results of stratified analyses assessing the influence of the interaction between *TEF*rs5758324 and peritraumatic distress on posttraumatic stress symptom (PTSS) severity following motor vehicle collision (MVC), sexual assault (SA) and major thermal burn injury (MThBI) trauma exposures. PTSS severity in individuals with low and high distress and the major or at least one copy of the minor allele at *TEF*rs5758324 shown in all panels. Specific cohort data shown as follows: **(A)** MVC study participants (*n* = 931). All participants in this study were African American. **(B)** SA study participants (*n* = 274). **(C)** African American individuals from the SA study (*n* = 46). **(D)** MThBI study participants (*n* = 68). **(E)** African American individuals from the MThBI study (*n* = 27). Outliers defined using Tukey criteria are represented by black dots. ^*^p-value < 0.05, ^#^*p*-value < 0.001.

Stratified analyses were performed to determine whether the direction and magnitude of the relationship between *TEF*rs5758324 and PTSS severity was similar in the three cohorts. Marginal means demonstrated that the direction of effect was similar across all three trauma exposures (Figure 1). Additionally, individuals with high distress and at least one copy of the *TEF*rs5758324 minor allele reported the highest PTSS severity compared to the other subgroups (Figure 1). This relationship persisted when adjusting for the time of trauma exposure, litigation status, and previous life trauma. Additionally, the effect of *TEF*rs5758324 and distress on PTSS severity following MVC did not seem to differ over the three posttraumatic timepoints measured (*F* = 0.51, *p* = 0.598). In the SA and MThBI cohorts, this difference was most pronounced among African American individuals. In sum, these data support the hypothesis that, *TEF*rs5758324 has a stress-dependent influence on PTSS severity, across a range of trauma exposures and ethnicities.

### *TEF*rs5758324 shows the strongest association with PTSS compared to other genetic variants in the surrounding region

**Figure 2:**
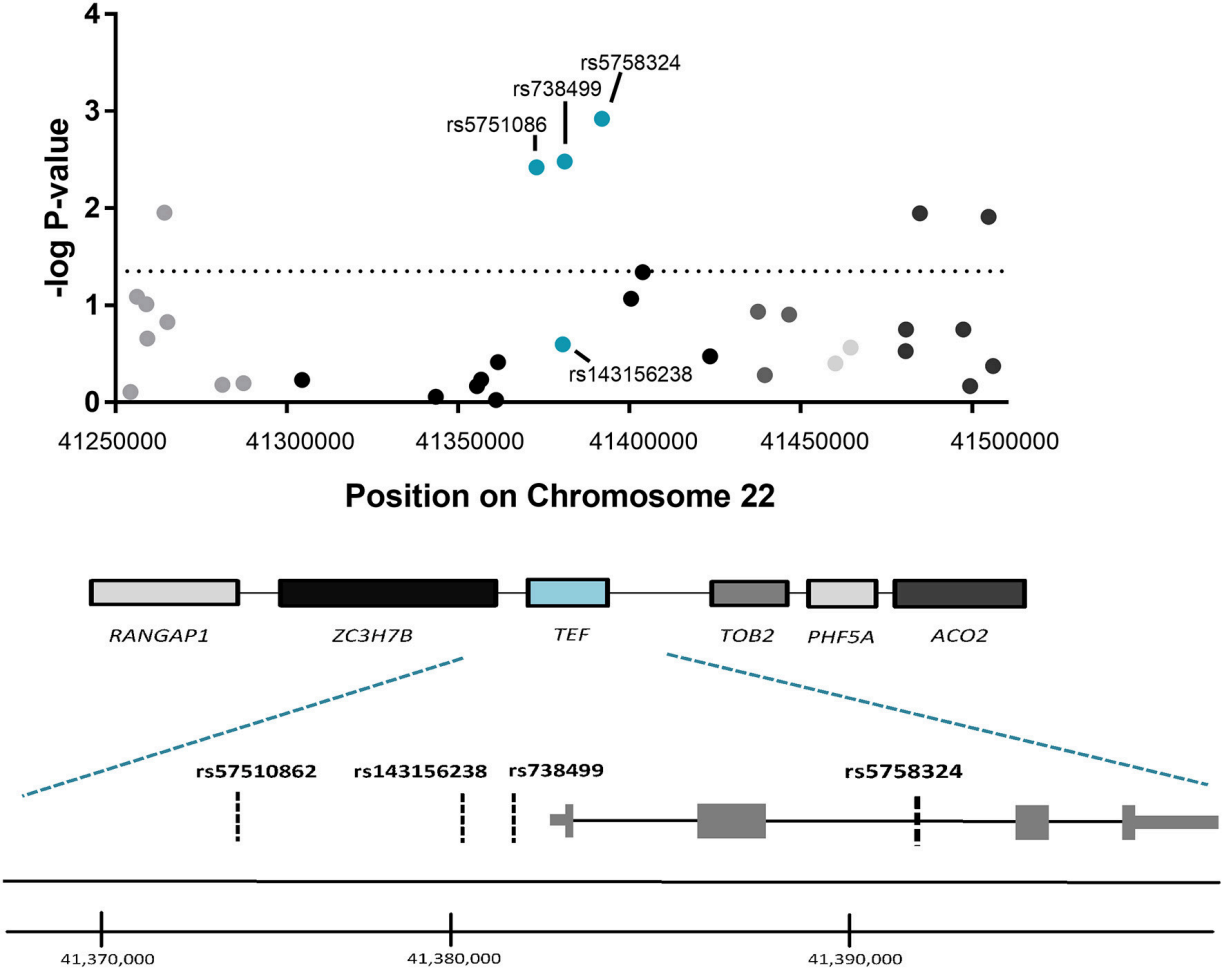
Association between genetic variants in the genomic region surrounding *TEF*rs5758324 and PTSS severity following motor vehicle collision (MVC) trauma. **(Top)** The –log *p*-values of the peritraumatic distress -dependent association between each genetic variant (represented by a dot on the graph) and PTSS severity following MVC were plotted vs. the location of each genetic variant on Chromosome 22. The horizontal dotted line indicates a significance threshold of *p* = 0.05. Corresponding genes mapping to the genomic region are indicated below the graph and shading/colors are coordinated between the dots representing genetic variants and the gene to which they map. **(Bottom)** Magnified schematic of the *TEF* gene indicating both isoforms for the *TEF* transcript, the relative location of exons and introns, and the relative location of each genetic variant assessed within the *TEF* gene. (*TEF* isoform 1: ENST00000266304.8 and *TEF* isoform 2: ENST00000406644.7). Genome coordinates refer to GRCh38/hg38 Assembly.

To determine whether the observed association for *TEF*rs5758324 is likely attributable to the *TEF* gene locus (vs. another gene in linkage disequilibrium with *TEF*rs5758324), we assessed for an association between PTSS and available genetic variants (i.e., variants in our genotyping array) within a 250 kb span surrounding *TEF*rs5758324. The strongest stress-dependent association with PTSS severity originated from three of the four genetic variants mapping to the *TEF* gene (rs5751086, *p* = 3.8 × 10^−3^; rs738499, *p* = 3.3 × 10^−3^; rs5758324, *p* = 1.2 × 10^−3^; Figure 2). These genetic variants are all located in intronic or upstream regions of *TEF* (Figure 2) and are in high linkage disequilibrium with each other (Supplementary Figure 3).

### *TEF*rs5758324 affects the relationship between *TEF* mRNA expression and PTSS severity

**Figure 3:**
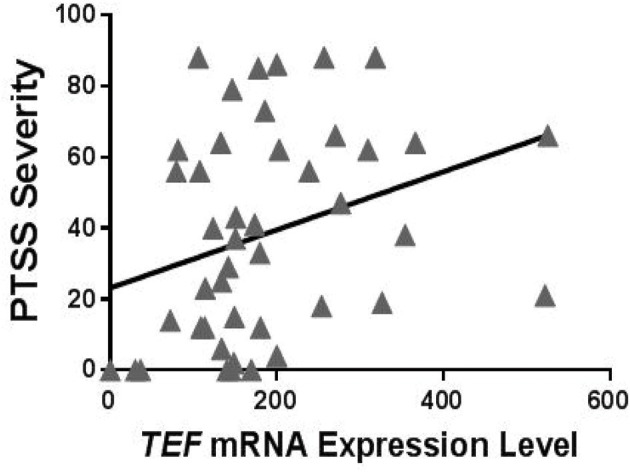
Relationship between circulating *TEF* mRNA expression levels and PTSS severity following motor vehicle collision (MVC) trauma in individuals who reported high peritraumatic distress in the early aftermath of trauma exposure and who had at least one copy of the *TEF*rs5758324 minor allele. Shown is representative data from PTSS levels measured 6 months following MVC (*n* = 50; Spearman's rho = 0.386, *p* = 0.009). No relationship between *TEF* mRNA and PTSS severity was observed for other subgroups of individuals: high peritraumatic distress and *TEF*rs5758324 major allele (*n* = 45; Spearman's rho = −0.007, *p* = 0.967), low peritraumatic distress and *TEF*rs5758324 minor allele (*n* = 42; Spearman's rho = −0.074, *p* = 0.642), low peritraumatic distress and *TEF*rs5758324 major allele (*n* = 38; Spearman's rho = −0.087, *p* = 0.604).

To evaluate potential functional effects of *TEF*rs5758324, we used RNA sequencing data available in a subset of individuals (*n* = 184) in the MVC cohort to assess the relationship between *TEF* mRNA expression levels and PTSS severity. To perform this analysis, we first stratified individuals with RNA data based on their peritraumatic distress level and the presence or absence of at least one copy of the *TEF*rs5758324 minor allele. In distressed individuals with at least one copy of the minor allele, we observed a statistically significant positive correlation between *TEF* mRNA and PTSS severity (Table 5, Figure 3). In the other three strata, there were no statistically significant correlations between *TEF* mRNA and PTSS severity (Table 5). These data indicate that the *TEF*rs5758324 minor allele strengthens the relationship between *TEF* RNA expression and subsequent PTSS severity in stressed individuals.

**Table 5:** Effect of stress and *TEF*rs5758324 on the correlation between *TEF* mRNA levels and PTSS severity following motor vehicle collision (MVC, *n* = 184).

**Distress level**	**Allele**	**Correlation between** ***TEF*** **mRNA and PTSS severity following MVC**	
		**Correlation coefficient^a^**	***p*-value**
Low distress	TT	−0.005	0.956
	TG/GG	−0.070	0.441
High distress	TT	0.048	0.599
	TG/GG	**0.183**	**0.035**

a *Spearman's rho*.

*Bold values indicate significance at the p < 0.05 threshold*.

## Discussion

In the present study, we searched among genetic variants in circadian pathway genes associated with neuropsychiatric disorders for variants that influence posttraumatic stress symptom severity after traumatic stress. We identified a genetic variant in the *TEF* gene, rs5758324, that had a stress-dependent influence on posttraumatic stress symptom severity across three different trauma exposures (MVC, SA, and MThBI) and across multiple ethnicities, such that individuals with peritraumatic distress and at least one copy of the minor allele had the highest levels of PTSS over time. Additional *TEF* variants in LD with *TEF*rs5758324 were also significantly associated with PTSS in a stress-dependent manner, suggesting that *TEF*rs5758324 might be an important risk locus for PTSS. Further, a strong positive relationship between *TEF* mRNA expression and PTSS severity was observed in individuals with high levels of peritraumatic distress and at least one copy of the *TEF*rs5758324 minor allele, suggesting that the variant may be functional.

*TEF* is one of several key regulatory transcription factors within the circadian rhythm pathway. *TEF*, along with several other key circadian-associated transcriptional activators, binds at D-box promoter sites to promote transcription of a number of clock-controlled genes as well as key genes in the core circadian feedback loop including *NR1D1, NR1D2, PER*, and *CRY* (89–91). How *TEF* polymorphisms might influence the pathogenesis of PTSS remains poorly understood. Such polymorphisms may affect sleep quality in the days or weeks after trauma, contributing to PTSS onset or preventing remittance via a variety of mechanisms (92, 93). In addition, *TEF* has been found to influence brain levels of serotonin and dopamine through the clock-controlled gene, *PDXK* (94). Future studies defining the role of *TEF* in PTSS pathogenesis, such as knock-out or gene silencing animal studies, or the evaluation of peritraumatic sleep patterns among distressed individuals with and without the *TEF*rs5758324 minor allele is warranted.

While previous studies have identified a relationship between polymorphisms in the *TEF* gene and PTSS-associated neuropsychiatric disorders, most focused on *TEF*rs738499, a polymorphism in high LD with *TEF*rs5758324 (D' = 0.987, *R*^2^ = 0.272). (This allele was associated with PTSS development in our discovery cohort, but did not replicate in SA and MThBI survivors). Previous literature has identified an association between *TEF*rs738499 and depression (74, 84–87) and sleep disturbances (88). Interestingly, an expanded study of the initial cohort that identified an association between *TEF*rs738499 and depression failed to replicate the relationship (73), suggesting that *TEF*rs738499 might not be a functional allele or might not have relevance to all populations or types of depression. The same expanded study that failed to replicate the association for *TEF*rs738499 identified an association at the trend-level for *TEF*rs5758324. Consistent with the hypothesis that *TEF*rs5758324 is functional, publicly available data from the GTEx consortium (95) indicates that in 44 cell lines/tissues examined, *TEF*rs5758324 is an expression quantitative trait locus (eQTL). Additionally, using data cataloged by Metamoodics (96), *TEF* mRNA is increased in the frontal cortex of individuals with neuropsychiatric disorders (though this online database did not distinguish genetic variant effects on mRNA expression).

We did not detect a main effect relationship between *TEF* alleles and PTSS outcomes similarly to previous studies. Instead, we detected a gene × environment interaction, where stressed individuals with at least one copy of the minor allele reported the highest PTSS severity. Such gene × stress interactions are being increasingly identified (97–99). For instance, the relationship between *FKBP5* alleles and PTSS/PTSS related neuropsychiatric outcomes have consistently been shown to be stress dependent (46, 47, 100–102). Future studies should consider such interactions when examining genetic associations between *TEF* or other circadian rhythm genes and neuropsychiatric disorders.

A number of limitations should be considered when interpreting this manuscript. First, this study used a candidate gene approach to test a specific hypothesis regarding the role of circadian rhythm genetic variants in predicting PTSS development. Many in the mental health field are moving away from candidate gene studies, in favor of GWAS approaches. However, given their ability to evaluate pathways with high pre-test probability, test for interactions, replicate results across multiple studies and ethnicities, and use multilayered data analyses to evaluate for evidence of functionality, we believe that they still have merit and utility to advance the field. Second, our functional assessment of *TEF*rs5758324 using mRNA expression analyses was limited to blood expression levels. *TEF* is widely expressed throughout the body, with highest expression in the brain. Therefore, it is possible that blood measurements of expression are not an accurate proxy for brain expression. A previous report showed that the transcriptome of some central nervous tissues and the blood overlap (103), suggesting that *TEF* blood expression might approximate nervous tissue expression. However, further studies are needed to directly address this possibility. Third, we only assessed association between four *TEF* variants and PTSS development. The use of targeted sequencing methods could provide further granulation of genetic variation across the *TEF* gene and enable us to pinpoint the most influential genetic variants in this genomic region (in addition to *TEF*rs5758324). Fourth, we did not examine whether additional factors related to stress or the circadian clock, such as stress hormone levels and/or sleep quality mediates the relationship between *TEF*rs5758324 and PTSS outcomes. However, such analyses would be of interest for future studies. Fifth, the list of candidate SNPs identified via literature search, that were analyzed for association with PTSS in the current study, was limited in scope due to the reliance on previous studies showing an association between a circradian rhythm associated gene and a PTSS related disorder. This study design resulted in an under sampling of SNPs in key circadian rhythm genes such as *CRY1* and *PER1*. In the future, higher-powered studies would benefit from assessing tagging SNPs across all circadian rhythm associated genes to identify additional circadian rhythm SNPs that might predict PTSS following trauma exposure. Sixth, we do not know from which blood cell component our RNA expression originated, thus limiting our ability to make inferences about the origin of *TEF* mRNA in this study. Seventh, only approximately one-third of African American individuals carry the *TEF*rs5758324 minor allele. In combination with African Americans comprising only a subset of the SA and MThBI cohorts, stratified analyses were likely underpowered. Finally, the genetic variant in *RORA*, rs8042149, which was identified in the first GWAS for PTSD (8) was unfortunately not included on our genotyping array, thus we were not able to assess whether this particular allele predicted PTSS in our trauma cohorts. Additionally, there was very little LD between our *RORA* variants and *RORA*rs8042149. Therefore, this study should not be considered a failed replication.

In conclusion, the above data, from multiple trauma exposures and across ethnicities, suggest that individuals with the *TEF*rs5758324 minor allele and high levels of peritraumatic distress experience more severe PTSS than individuals with the *TEF*rs5758324 major allele. Further, the above data provide preliminary evidence that *TEF*rs5758324 is functional. Further studies are needed to elucidate potential mechanisms mediating these relationships, as improved understanding of such mechanisms could contribute to improved PTSS prevention and treatment.

## Ethics statement

This study was carried out in accordance with the recommendations of the Institutional Review Board at each participating hospital with written informed consent from all subjects. All subjects gave written informed consent in accordance with the Declaration of Helsinki. The protocol was approved by the Institutional Review Board at the data coordinating center and at each enrolling hospital.

## Author's note

Scientific Meeting Presentation: Society for Biological Psychiatry Annual Meeting, May 2017.

## Author contributions

SL and CZ: conceived the manuscript; SL, YP, MM, CZ, LJ, CR, JB, MC, and AT: performed analyses and made figures/tables; SL and SM: contributed to manuscript design and writing; JS, MK, PH, CL, TD, ED, KB, ML, JWS, and BC: assisted with study design and were responsible for data collection at individual ED sites.

### Conflict of interest statement

The authors declare that the research was conducted in the absence of any commercial or financial relationships that could be construed as a potential conflict of interest. The handling Editor declared a shared affiliation, though no other collaboration, with several of the authors SL, YP, MM, JS, CZ, LJ, CR, JB, MC, AT, BC, SM, at the time of the review.

## Abbreviations

VC: motor vehicle collision
SA: sexual assault
MThBI: major thermal burn injury
ED: emergency department
DNA: deoxyribonucleic acid
RNA: ribonucleic acid
*TEF*: thyrotroph embryonic factor
PTSS: posttraumatic stress symptoms
GWAS: genome wide association study.
