# Supplementary material for: Evaluation of the Association Between Genetic Variants in Circadian Rhythm Genes and Posttraumatic Stress Symptoms Identifies a Potential Functional Allele in the Transcription Factor TEF
Source: Front Psychiatry. 2018 Nov 15;9:597. doi: 10.3389/fpsyt.2018.00597 (PMC6249322; doi:10.3389/fpsyt.2018.00597)
Supplement: Supplementary file 1 [file Table_1.DOCX]

| **Supplementary Table 1.** Minor allele frequency (MAF) and Hardy Weinberg equilibrium (HWE) values for genetic variants assessed in the motor vehicle collision cohort. | | | | |
| --- | --- | --- | --- | --- |
| **Gene Symbol** | **Genetic variant** | **Alleles** | **MAF** | **HWE** |
| *ARNTL; BMAL1* | [rs7107287](http://www.ncbi.nlm.nih.gov/projects/SNP/snp_ref.cgi?rs=7107287) | C/A | 0.22 | 0.78 |
|  | [rs1982350](http://www.ncbi.nlm.nih.gov/projects/SNP/snp_ref.cgi?rs=1982350) | G/A | 0.47 | 0.55 |
|  | [rs11022778](http://www.ncbi.nlm.nih.gov/projects/SNP/snp_ref.cgi?rs=11022778) | A/C | 0.21 | 1.00 |
|  | [rs969485](http://www.ncbi.nlm.nih.gov/projects/SNP/snp_ref.cgi?rs=969485) | A/G | 0.48 | 0.74 |
| *CLOCK* | [rs534654](http://www.ncbi.nlm.nih.gov/projects/SNP/snp_ref.cgi?rs=534654) | G/A | 0.20 | 0.18 |
|  | [rs1801260](http://www.ncbi.nlm.nih.gov/projects/SNP/snp_ref.cgi?rs=1801260) | A/G | 0.17 | 0.91 |
| *NPAS2* | rs1562313 | G/A | 0.28 | 0.42 |
|  | [rs12622050](http://www.ncbi.nlm.nih.gov/projects/SNP/snp_ref.cgi?rs=12622050) | G/A | 0.29 | 0.20 |
|  | [rs2305159](http://www.ncbi.nlm.nih.gov/projects/SNP/snp_ref.cgi?rs=2305159) | C/A | 0.45 | 0.29 |
|  | [rs6740935](http://www.ncbi.nlm.nih.gov/projects/SNP/snp_ref.cgi?rs=6740935) | G/A | 0.40 | 0.41 |
| *PER2* | [rs6431590](http://www.ncbi.nlm.nih.gov/projects/SNP/snp_ref.cgi?rs=6431590) | G/A | 0.34 | 0.51 |
| *PER3* | [rs10462018](http://www.ncbi.nlm.nih.gov/projects/SNP/snp_ref.cgi?rs=10462018) | G/A | 0.15 | 0.46 |
|  | [rs228642](http://www.ncbi.nlm.nih.gov/projects/SNP/snp_ref.cgi?rs=228642) | G/A | 0.37 | 0.21 |
| *RORA* | [rs12912233](http://www.ncbi.nlm.nih.gov/projects/SNP/snp_ref.cgi?rs=12912233) | G/A | 0.24 | 0.86 |
|  | [rs4774388](http://www.ncbi.nlm.nih.gov/projects/SNP/snp_ref.cgi?rs=4774388) | T/C | 0.22 | 0.45 |
|  | [rs2414680](http://www.ncbi.nlm.nih.gov/projects/SNP/snp_ref.cgi?rs=2414680) | A/G | 0.25 | 0.60 |
|  | [rs16943472](http://www.ncbi.nlm.nih.gov/projects/SNP/snp_ref.cgi?rs=16943472) | G/C | 0.23 | 0.51 |
|  | [rs4775351](http://www.ncbi.nlm.nih.gov/projects/SNP/snp_ref.cgi?rs=4775351) | A/G | 0.46 | 0.84 |
|  | [rs8023563](http://www.ncbi.nlm.nih.gov/projects/SNP/snp_ref.cgi?rs=8023563) | A/T | 0.47 | 0.06 |
|  | rs12906588 | C/A | 0.47 | 1.00 |
|  | rs809736 | A/G | 0.11 | 0.08 |
|  | rs782931 | A/G | 0.39 | 1.00 |
|  | rs13329238 | A/C | 0.46 | 0.11 |
|  | rs9302215 | G/A | 0.31 | 0.70 |
|  | rs11071557 | A/G | 0.33 | 0.71 |
|  | rs12915776 | G/A | 0.23 | 0.93 |
|  | rs8041466 | G/A | 0.35 | 0.94 |
|  | rs34720147 | G/A | 0.17 | 0.36 |
| *RORB* | [rs7022435](http://www.ncbi.nlm.nih.gov/projects/SNP/snp_ref.cgi?rs=7022435) | G/A | 0.20 | 0.18 |
| *TEF* | [rs738499](http://www.ncbi.nlm.nih.gov/projects/SNP/snp_ref.cgi?rs=738499) | T/G | 0.15 | 0.05 |
|  | [rs5758324](http://www.ncbi.nlm.nih.gov/projects/SNP/snp_ref.cgi?rs=5758324) | T/G | 0.29 | 1.00 |
| *TIMELESS* | [rs11171856](http://www.ncbi.nlm.nih.gov/projects/SNP/snp_ref.cgi?rs=11171856) | G/A | 0.42 | 0.79 |
| *RANGAP1* | rs2229755 | C/G | 0.05 | 0.52 |
|  | rs114673061 | G/C | 0.01 | 1.00 |
|  | rs139513 | G/A | 0.28 | 0.19 |
|  | rs6002301 | G/A | 0.10 | 0.28 |
|  | rs386821456 | G/A | 0.21 | 1.00 |
|  | rs2235852 | C/A | 0.25 | 0.08 |
|  | rs139533 | G/A | 0.33 | 0.71 |
|  | rs9611543 | C/A | 0.22 | 1.00 |
| *ZC3H7B* | rs73885767 | G/A | 0.03 | 0.26 |
|  | rs73416822 | G/A | 0.10 | 1.00 |
|  | rs35055668 | G/A | 0.04 | 0.21 |
|  | rs12484074 | G/A | 0.09 | 0.41 |
| *ZC3H7B, TOB2* | rs4822024 | G/A | 0.16 | 0.01 |
| *TEF* | rs5751086 | A/G | 0.47 | 0.01 |
|  | rs143156238 | A/G | 0.01 | 0.13 |
| *TOB2* | rs2011790 | A/G | 0.06 | 0.01 |
|  | rs202654 | G/A | 0.35 | 0.88 |
|  | rs58347890 | C/A | 0.04 | 0.31 |
| *PHF5A* | rs17002469 | G/A | 0.01 | 0.12 |
|  | rs10483211 | A/C | 0.13 | 0.48 |
| *ACO2* | rs58744979 | C/A | 0.08 | 0.50 |
|  | rs60239624 | T/A | 0.05 | 0.47 |
|  | rs5751114 | A/G | 0.47 | 0.95 |
|  | rs78595535 | A/G | 0.04 | 0.33 |
|  | rs2076196 | G/A | 0.03 | 1.00 |
|  | rs7289322 | A/C | 0.35 | 0.83 |
|  | rs527246349 | C/A | 0.04 | 0.65 |
