# Supplementary material for: Evaluation of the Association Between Genetic Variants in Circadian Rhythm Genes and Posttraumatic Stress Symptoms Identifies a Potential Functional Allele in the Transcription Factor TEF
Source: Front Psychiatry. 2018 Nov 15;9:597. doi: 10.3389/fpsyt.2018.00597 (PMC6249322; doi:10.3389/fpsyt.2018.00597)
Supplement: Supplementary file 2 [file Table_2.DOCX]

| **Supplementary Table 2.** Minor allele frequency (MAF) and Hardy Weinberg equilibrium (HWE) values for genetic variants assessed in the sexual assault (SA) and major thermal burn injury (MThBI) cohorts. | | | | | | |
| --- | --- | --- | --- | --- | --- | --- |
| **Gene Name** | **SNP** | **Alleles** | **SA Cohort** | | **MThBI Cohort** | |
|  |  |  | **MAF** | **HWE** | **MAF** | **HWE** |
| *RORB* | rs7022435 | G/A | 0.19 | 0.44 | 0.24 | 0.24 |
| *BMAL1* | rs969485 | A/G | 0.38 | 0.61 | 0.29 | 0.84 |
| *RORA* | rs4774388 | T/C | 0.19 | 0.69 | 0.18 | 1.00 |
| *NPAS2* | rs12622050 | G/A | 0.26 | 0.16 | 0.17 | 0.77 |
| *TEF* | rs5758324 | T/G | 0.41 | 0.05 | 0.40 | 1.00 |
|  | rs738499 | T/G | 0.22 | 0.38 | 0.27 | 0.13 |
