# Supplementary material for: Evaluation of the Association Between Genetic Variants in Circadian Rhythm Genes and Posttraumatic Stress Symptoms Identifies a Potential Functional Allele in the Transcription Factor TEF
Source: Front Psychiatry. 2018 Nov 15;9:597. doi: 10.3389/fpsyt.2018.00597 (PMC6249322; doi:10.3389/fpsyt.2018.00597)
Supplement: Supplementary file 4 [file Table_4.DOCX]

| **Supplementary Table 3.** Baseline characteristics of MVC study participants stratified by *TEF*rs5758324 allele status | | | | |
| --- | --- | --- | --- | --- |
| **Characteristic** | **MVC^a^** | **rs5758324**  **TT** | **rs5758324**  **TG/GG** | **t-test**  **(p-value)** |
| Enrolled, n | 930 | 454 | 455 |  |
| Age, years, mean (SD) | 35.1 (12.7) | 34.8 (12.3) | 35.3 (12.9) | -0.60 (0.55) |
| Females, n (%) | 578 (62.2) | 284 (62.6) | 282 (62.0) | -0.18 (0.86) |
| African American, n (%) | 930 (100) | 454 (100) | 455 (100) |  |
| Education, n (%) |  |  |  |  |
| 0-11 yrs | 71 (7.6) | 36 (8.0) | 43 (9.5) | -0.81 (0.42) |
| High School | 290 (31.2) | 131 (28.9) | 154 (34.2) | -1.62 (0.10) |
| Post-High School | 42 (4.5) | 21 (4.6) | 20 (4.4) | 0.17 (0.87) |
| Some college | 338 (36.3) | 185 (40.8) | 145 (32.2) | 2.78 (0.005) |
| College graduate | 137 (14.7) | 66 (14.6) | 67 (14.9) | -0.08 (0.94) |
| Post-graduate studies | 36 (3.9) | 14 (3.1) | 22 (4.9) | 3.21 (0.001) |
