# Supplementary figures and images for: Evaluation of the Association Between Genetic Variants in Circadian Rhythm Genes and Posttraumatic Stress Symptoms Identifies a Potential Functional Allele in the Transcription Factor TEF
Source: Front Psychiatry. 2018 Nov 15;9:597. doi: 10.3389/fpsyt.2018.00597 (PMC6249322; doi:10.3389/fpsyt.2018.00597)

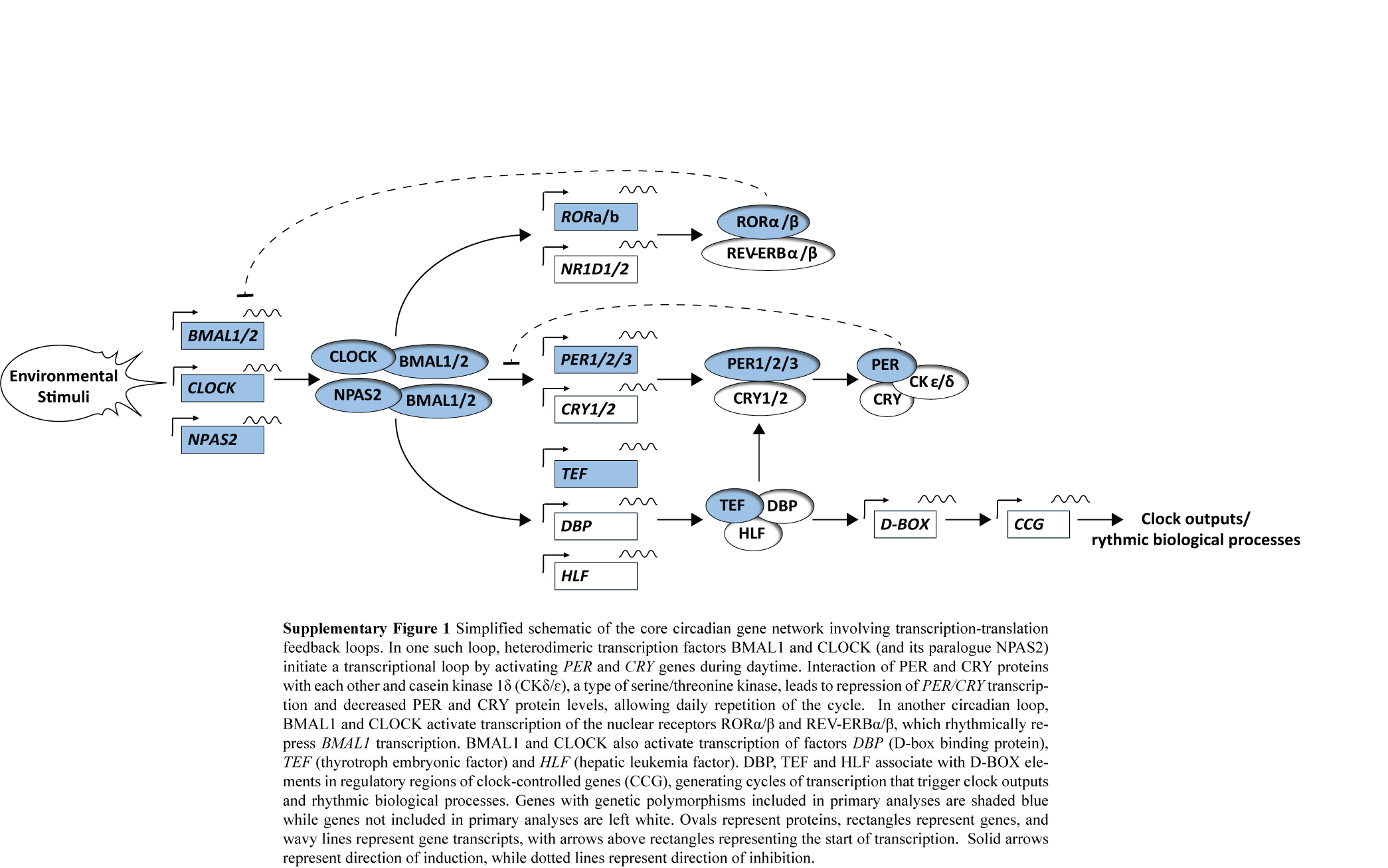

Supplement: Supplementary file 5 [file Image_1.TIF]

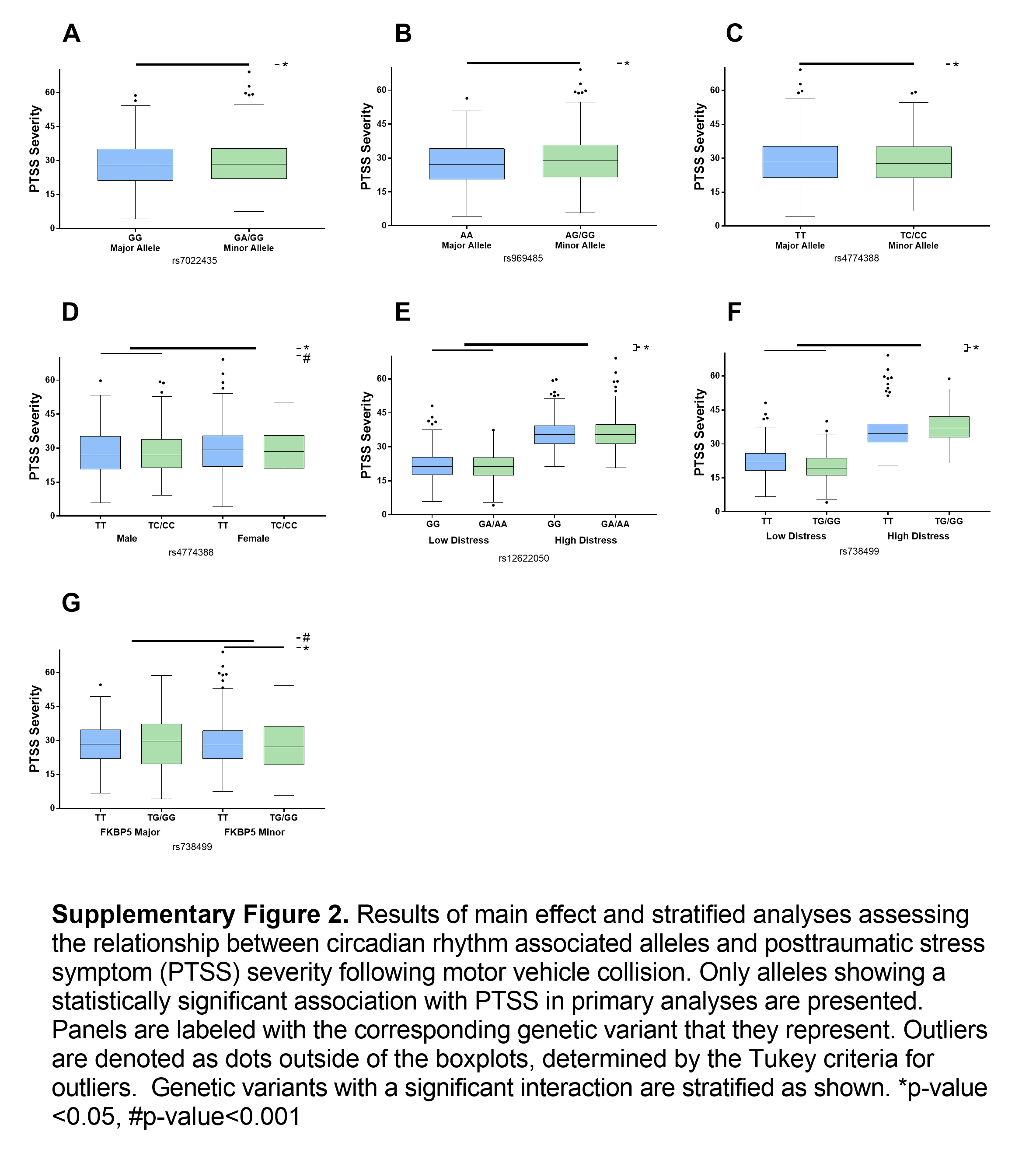

Supplement: Supplementary file 6 [file Image_2.TIF]

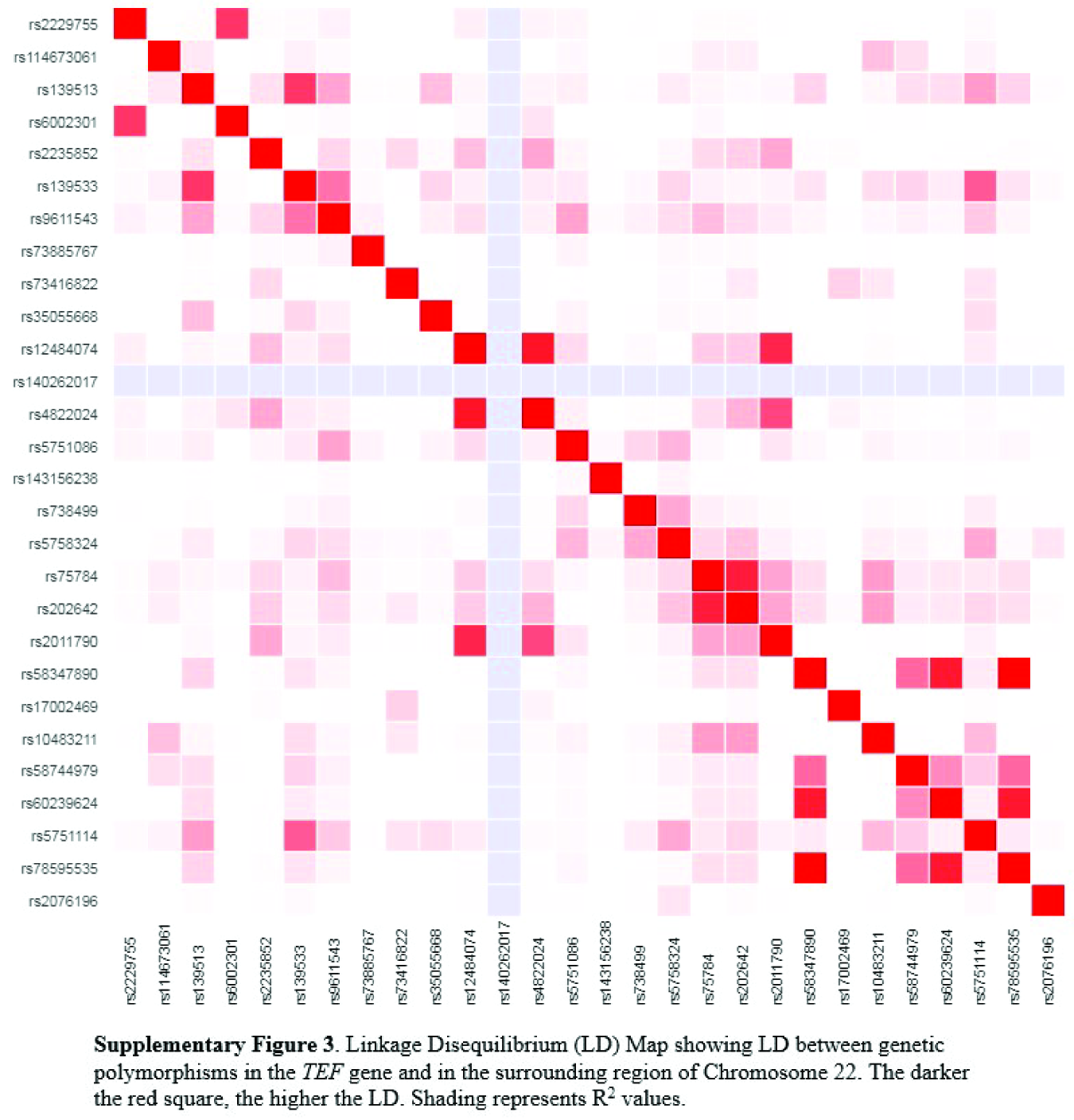

Supplement: Supplementary file 7 [file Image_3.tif]
